# TRAF6 Regulates the Immunosuppressive Effects of Myeloid-Derived Suppressor Cells in Tumor-Bearing Host

**DOI:** 10.3389/fimmu.2021.649020

**Published:** 2021-02-25

**Authors:** Ge Song, Yue Zhang, Jie Tian, Jie Ma, Kai Yin, Huaxi Xu, Shengjun Wang

**Affiliations:** ^1^Department of Laboratory Medicine, Affiliated People's Hospital, Jiangsu University, Zhenjiang, China; ^2^Institute of Laboratory Medicine, Jiangsu Key Laboratory for Laboratory Medicine, Jiangsu University School of Medicine, Zhenjiang, China; ^3^Department of General Surgery, Affiliated Hospital of Jiangsu University, Zhenjiang, China

**Keywords:** myeloid-derived suppressor cells, TRAF6, STAT3, tumor immunology, tumor microenvironment

## Abstract

Myeloid-derived suppressor cells (MDSCs) are immature heterogeneous cells derived from the bone marrow and they are the major component of the tumor-induced immunosuppressive environment. Tumor necrosis factor receptor-associated factor 6 (TRAF6), an E3 ubiquitin ligase, catalyzes the polyubiquitination of target proteins. TRAF6 plays a critical role in modulating the immune system. However, whether TRAF6 is involved in the regulation of MDSCs has not been thoroughly elucidated to date. In this study, we found that the expression of TRAF6 in MDSCs derived from tumor tissue was significantly upregulated compared with that of MDSCs from spleen of tumor-bearing mice. Knockdown of TRAF6 remarkably attenuated the immunosuppressive effects of MDSCs. Mechanistically, TRAF6 might improve the immunosuppression of MDSCs by mediating K63-linked polyubiquitination and phosphorylation of signal transducer and activator of transcription 3 (STAT3). Additionally, it was discovered that the accumulation of MDSCs was abnormal in peripheral blood of lung cancer patients. TRAF6 and arginase 1 were highly expressed in MDSCs of patients with lung cancer. Taken together, our study demonstrated that TRAF6 participates in promoting the immunosuppressive function of MDSCs and provided a potential target for antitumor immunotherapy.

## Introduction

The tumor microenvironment provides a suitable environment for the survival and development of tumor cells. There are a large number of cytokines and chemokines in the tumor microenvironment that recruit immunosuppressive cells to accumulate in local tumors and promote tumor immune escape (1–3). Myeloid-derived suppressor cells (MDSCs) are crucial immunosuppressive cells in the tumor microenvironment, which have powerful suppressive effects (4–6). In mice, MDSCs are immature myeloid cells that co-express CD11b and Gr1, which are further divided into 2 subsets: CD11b^+^Ly6G^+^Ly6C^low^ polymorphonuclear MDSCs (PMN-MDSCs) and CD11b^+^Ly6G^−^Ly6C^hi^ mononuclear MDSCs (M-MDSCs) according to the expression of LY6G and LY6C (7–10). However, human MDSCs have a more complex phenotype and are usually characterized by CD11b^+^CD33^+^HLA-DR^low/−^ expression (9, 11). In tumor-bearing mice, the proportion of MDSCs is significantly increased compared with that in wild type mice. MDSCs reach 20 to 40% in the spleen in tumor-bearing mice, and the massive aggregation of MDSCs in tumor tissue is particularly obvious (12). Studies have shown that the immunosuppressive function of MDSCs derived from tumor tissue was stronger than that of spleen-derived MDSCs from tumor-bearing mice (13–15).

Accumulating evidence has indicated that the suppressive function of MDSCs is correlated with the high production of arginase 1 (Arg1), inducible nitric oxide synthase (iNOS) and reactive oxygen species (ROS), which can inhibit T cell proliferation and antitumor responses (16–18). The two subsets of MDSCs exert immunosuppressive effects in different ways. Although both PMN-MDSCs and M-MDSCs highly express Arg1, the expression of iNOS and ROS are notably different (19, 20). Signal transducer and activator of transcription 3 (STAT3) is one of the key transcription factors regulating the expansion, activation and function of MDSCs (21–24). Abnormal activation of STAT3 signaling hinders the normal differentiation of myeloid cells, leading to the expansion and activation of MDSCs (25–27). STAT3 activation is mainly affected by posttranslational modifications. In addition to phosphorylation modification, ubiquitination also plays a critical role in modulating the activity of STAT3 (28, 29).

Tumor necrosis factor receptor-associated factor 6 (TRAF6) is an important member of the TRAF family (30). Unlike other TRAF members, TRAF6 is both an adaptor protein and E3 ubiquitin ligase (31–33). As a non-conventional E3 ubiquitin ligase, TRAF6 catalyzes the K63-linked polyubiquitination of target proteins further affecting functions. Studies have shown that the E3 ubiquitin ligase TRAF6 binds to STAT3 (28, 34). Moreover, TRAF6 promotes STAT3 phosphorylation by mediating the K63-linked polyubiquitination of STAT3 (29). However, whether TRAF6 affects the function of MDSCs by mediating STAT3 activity is unclear.

In this study, we explored the role of TRAF6 in modulating the immunosuppressive function of MDSCs and elaborated the potential molecular mechanism. Our research pointed out that the expression of TRAF6 is significantly increased in MDSCs derived from the tumor tissue of tumor-bearing mice. TRAF6 promoted the immunosuppressive function of MDSCs by mediating the activation of STAT3. These data elucidate a novel mechanism of the regulation of MDSCs in the tumor microenvironment and propose new ideas for therapeutic strategies targeting TRAF6.

## Methods

### Cell Line, Mice, and Tumor Models

Male C57BL/6 mice, aged 6–8 weeks, were purchased from the Laboratory Animal Center of Jiangsu University (Zhenjiang, China). Murine Lewis lung carcinoma (LLC) cells were obtained from the Cell Bank of Shanghai Institutes for Biological Sciences (Shanghai, China). The cells were cultured in DMEM (Gibco, Carlsbad, CA) with 10% fetal calf serum (Gibco, Carlsbad, CA) at 37°C in a humidified 5% CO_2_ atmosphere. 1 × 10^6^ LLC cells were injected subcutaneously into the mice to establish tumor-bearing mouse model. All animal experiments were approved by the Committee on the Use of Live Animals in Research and Teaching of Jiangsu University.

### Preparation of Single-Cell Suspensions

The murine spleen was ground and treated with ACK buffer. After centrifugation, PBE buffer was added to obtain a spleen cell suspension. In addition, the tumor tissue was stripped and cut into pieces, and then collagenase, hyaluronidase and DNase I (Sigma-Aldrich, St. Louis, MO) were used to digest the tissue in a water bath at 37°C for 2 h. The filtrate was collected through a 70 μm cell strainer. After centrifugation, PBE buffer was added to obtain single-cell suspensions.

### Isolation of MDSCs and CD4^+^ T Cells

Murine Gr1^+^ CD11b^+^ MDSCs were isolated using a mouse MDSC kit (Miltenyi Biotec, Auburn, CA) according to the manufacturer's instructions. To improve the purity of MDSCs isolated from tumor tissues, enriched MDSCs were subsequently isolated using flow cytometry (FCM).

Simultaneously, murine CD4^+^ T cells were isolated from the spleens of wild-type C57BL/6 mice via using a mouse CD4^+^ T cell isolation kit (Miltenyi Biotec, Auburn, CA). The purity of MDSCs and CD4^+^ T cells were determined by FCM.

### Flow Cytometry

Single-cell suspensions were stained with relevant fluorochrome-conjugated anti-mouse/human CD11b, anti-mouse Gr-1, Ly6G, and Ly6C antibodies (Biolegend, San Diego, CA) and anti-human-CD33 and HLA-DR antibodies (eBioscience, San Diego, CA). To examine cytotoxic T lymphocytes (CTLs) and T helper 1 (Th1) cells, single-cell suspensions derived from tumor tissues of tumor-bearing mice were treated with 1 μg/mL ionomycin, 2 ng/mL monensin (eBioscience, San Diego, CA), and 50 ng/mL PMA (Sigma-Aldrich, St. Louis, MO) for 5 h. After resuspending in PBS, the cells were stained with anti-mouse CD3e, anti-mouse CD8a or anti-mouse CD4 mAb (eBioscience, San Diego, CA). The cells were incubated at 4°C for 30 min, fixed, permeabilized, and stained with anti-mouse IFN-γ mAbs (BD Pharmingen™) according to the instructions in the intracellular cytokine staining kit (eBioscience, San Diego, CA). Flow cytometry (BD FACSCalibur) was used to determine the proportion of cells.

### Quantitative Real-Time PCR (qRT-PCR)

Total RNA was extracted by using TRIzol reagent (Invitrogen, Carlsbad, CA) and reverse-transcribed to cDNA with a PrimeScript RT reagent kit (Takara, Osaka, Japan) according to the manufacturer's instructions. Quantitative PCR was performed by using SYBR Premix Ex Taq (Tli RNaseH Plus) (Takara, Osaka, Japan). The primer sequences are listed in Table 1.

**Table 1:** The gene primer sequences.

**Gene**	**Primer sequence**
Arg1	Forward: 5′-GCTGGTCTGCTGGAAAAACTT-3′
	Reverse: 5′-AGGGGAGTGTTGATGTCAGTGT-3′
iNOS	Forward: 5′-GAGCCCTCAGCAGCATCCAT-3′
	Reverse: 5′-GGTGAGGGCTTGGCTGAGTG-3′
18S	Forward: 5′-TCCGGAGAGGGAGCCTGAGA-3′
	Reverse: 5′-GCACCAGACTTGCCCTCCAA-3′
TRAF6	Forward: 5′-TGCTTTGCGTCCGTGCGATG-3′
	Reverse: 5′-GGGTCCGAATGGTCCGTTTG-3′
β-actin	Forward: 5′-AGCCATGTACGTAGCCATCC-3′
	Reverse: 5′-GCTGTGGTGGTGAAGCTGTA-3′

### Immunoprecipitation and Western Blotting

MDSCs were lysed by immunoprecipitation cell lysis buffer. After centrifugation, the supernatant was collected and incubated with anti-STAT3 Abs (Santa Cruz Biotechnology, Santa Cruz, CA) or IgG Abs (Cell Signaling Technology, Beverly, MA) for 30 min at 4°C. Then, Protein A/G plus-agarose beads (Santa Cruz Biotechnology, Santa Cruz, CA) were added and blended on a shaker overnight at 4°C. After being washed 3 times, the lysates were boiled in SDS-PAGE protein loading buffer. TRAF6 and STAT3 protein were detected by Western blotting.

Proteins extracted from cells were denatured and subsequently separated by SDS-PAGE. Then, the proteins were transferred to PVDF membranes (Bio-Rad, Hercules, CA), and the membranes were incubated in primary antibodies overnight at 4°C, followed by incubation with HRP-conjugated secondary antibodies. Detection was performed by using LAS4000 chemiluminescence gel imaging and analysis system (Champion Chemical, Whittier, CA). Rabbit anti-TRAF6 mAb, rabbit anti-ubiquitin (linkage-specific K63) mAb, and HRP-conjugated goat anti-rat IgG Ab were purchased from Abcam (Cambridge, UK). Rabbit anti–p-STAT3 (Y705) mAb and rat anti-β-actin mAb were obtained from Cell Signaling Technology (Beverly, MA).

### Transfection

Tumor tissue-derived MDSCs were cultured in 24-well plates with RPMI 1640 medium containing 10% FBS. MDSCs were transfected with TRAF6 siRNA or negative control siRNA using Lipofectamine ™ 2,000 transfection reagent (Invitrogen, Carlsbad, CA) according to the manufacturer's instructions. The siRNA transfection efficiency was determined by qRT-PCR and Western blotting. TRAF6 siRNA and negative control siRNA were purchased from RiboBio (Guangzhou, China).

### Assessment of MDSCs Suppressive Activity

In order to determine the immunosuppressive function of MDSCs, the sorted MDSCs were co-cultured with CFSE-labeled splenic CD4^+^ T cells in the study. Splenic CD4^+^ T cells were stained with fluorescent dye CFSE (5 μM, Invitrogen) for 10 min at 37°C protected from light. RPMI 1640 medium (Gibco, Carlsbad, CA) containing 10% fetal calf serum (Gibco, Carlsbad, CA) was added to wash the cell pellets for 3 times. Sorted MDSCs were co-cultured with CFSE-stained CD4^+^ T cells at a ratio of 1:1 in 96-well round-bottomed plates (Costar, Corning, NY) in the presence of anti-CD3 mAbs and anti-CD28 mAbs (Biolegend, San Diego, CA). The cells were incubated in RPMI 1640 medium supplemented with 10% fetal calf serum at 37°C in a humidified 5% CO2 atmosphere for 72 h protected from light. The proliferation of CD4^+^ T cells was detected by flow cytometry at 488 nm excitation light to determinate the suppressive activity of MDSCs.

### Measurement of Arginase 1 Activity and NO Content

MDSCs were lysed with an appropriate amount of RIPA buffer for 30 min, and the lysate supernatant was collected after centrifugation. A QuantiChrom arginase assay kit (BioAssay systems, Hayward, CA) was used to determine the Arg1 activity in the lysate supernatant.

The content of NO was measured by the Griess reagent system kit (Promega, Madison, WI) according to the manufacturer's instructions.

### *In vivo* Experiments

To study the effect of TRAF6 on the immunosuppressive function of MDSCs *in vivo*, C57BL/6 mice were divided into siRNA control group and siTRAF6-MDSC group. The mice in the siRNA control group were subcutaneously injected with 1 × 10^6^ MDSCs transfected with siNC mixed with 1 × 10^6^ LLC cells, and the mice in the siTRAF6-MDSC group were subcutaneously injected with 1 × 10^6^ MDSCs transfected with siTRAF6 mixed with 1 × 10^6^ LLC cells. We constantly monitored the length and width of the tumor, and the tumor volume was calculated using the formula V = 1/2 × a^2^ × b (“a” represents the width and “b” represents the length). The mice were sacrificed on the 28th day after inoculation with LLC cells and MDSCs. The proportions of Th1 cells and CTLs from tumor tissue were analyzed by FCM.

### Patients and Sampling

Fresh peripheral blood samples from 33 patients with lung cancer and healthy controls were collected at the Affiliated People's Hospital, Jiangsu University. The peripheral blood mononuclear cells (PBMCs) were isolated by density-gradient centrifugation via using Ficoll-Hypaque solution (Haoyang Biological Technology Co.) and subsequently analyzed by flow cytometry. The protocol was approved by the Ethics Committee of the Affiliated People's Hospital of Jiangsu University (Zhenjiang, China). Written informed consent was obtained from all patients before study enrollment.

### Statistical Analysis

The experimental data are expressed as the mean ± SD. Student's *t*-test and ANOVA were used to determine significant differences. Correlations were determined by the Spearman correlation coefficient. Differences were considered significant at a *p* < 0.05.

## Results

### TRAF6 Is Highly Expressed in MDSCs Derived From Tumor Tissue of Tumor-Bearing Mice

**Figure 1:**
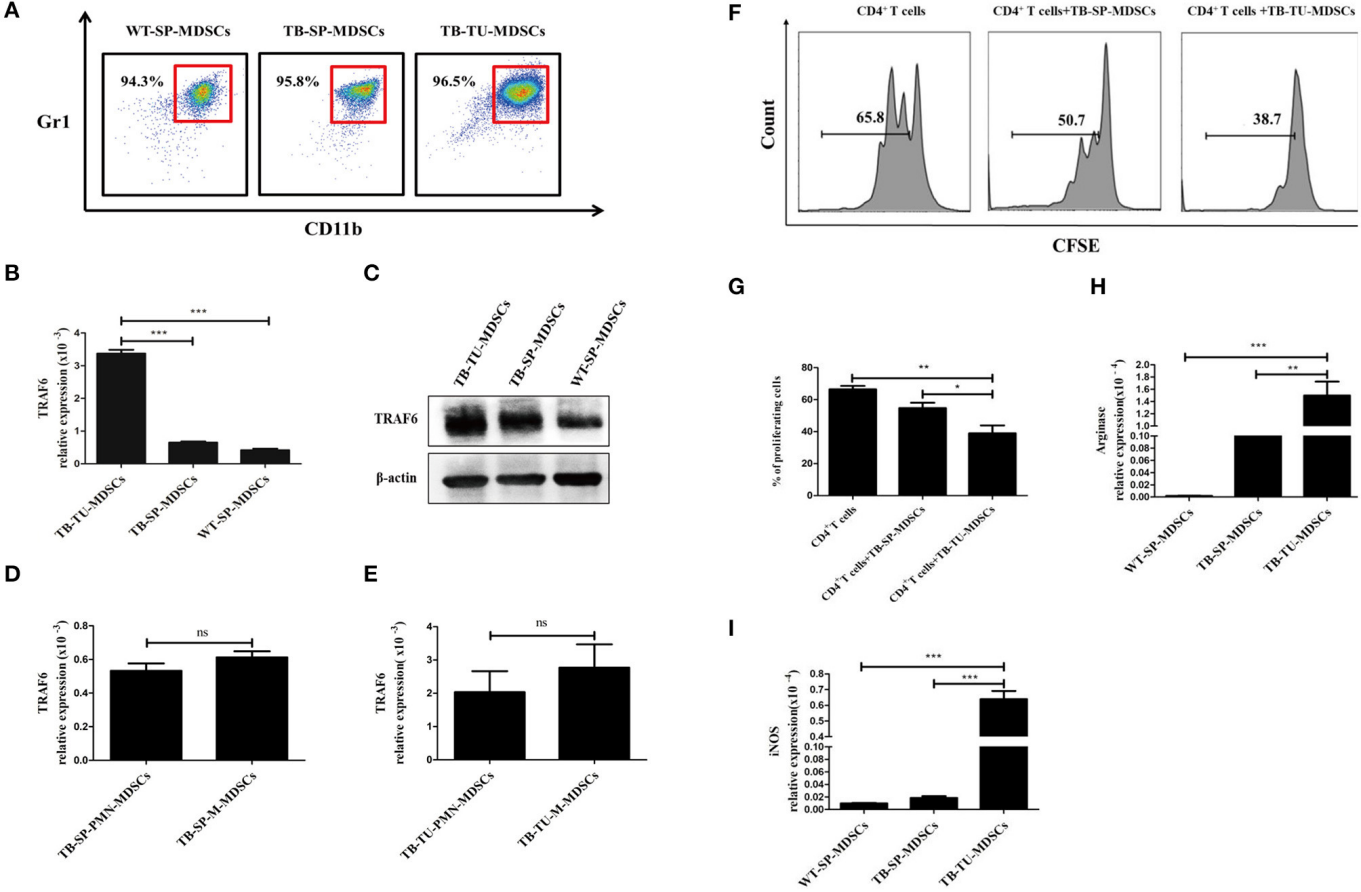
TRAF6 is highly expressed in MDSCs derived from the tumor tissue of tumor-bearing mice. Approximately 1 × 10^6^ LLC cells were s.c. injected in the backs of C57BL/6 mice for 28 d to establish a tumor-bearing (TB) mouse model. MDSCs were isolated by immunomagnetic beads from the spleens of TB mice, the tumor tissue of TB mice or the spleens of wild-type (WT) mice. **(A)** The purity of the isolated MDSCs was determined using flow cytometry via the detection of the CD11b^+^Gr1^+^ phenotype. The expression of TRAF6 in MDSCs derived from different sources was determined by qRT-PCR **(B)** or Western blotting **(C)**. The mRNA expression of TRAF6 in PMN-MDSCs and M-MDSCs derived from the spleen **(D)** or tumor tissue **(E)**. **(F)** CFSE-labeled CD4^+^ T cells were co-cultured with MDSCs derived from the spleen or tumor tissue in the presence of CD3 and CD28 stimulation. After 72 h, the proliferation of CD4^+^ T cells was tested via flow cytometry. **(G)** Statistical analyses of the percentage of proliferating CD4^+^ T cells co-cultured with MDSCs derived from the spleen or tumor tissue of TB mice. The mRNA expression levels of Arg1 **(H)** and iNOS **(I)** in MDSCs were measured by qRT-PCR. ***p < 0.001, **p < 0.01, *p < 0.05; ns, no significance; TB-TU-MDSCs, MDSCs derived from the tumor tissue of tumor-bearing mice; TB-SP-MDSCs, MDSCs derived from the spleens of tumor-bearing mice; WT-SP-MDSCs, MDSCs derived from the spleens of wild-type mice.

To investigate whether TRAF6 is involved in regulating MDSCs, we evaluated the expression of TRAF6 in MDSCs. The purity of MDSCs isolated from the spleen or tumor tissue of mice was >90% (Figure 1A). Compared with that of MDSCs from the spleens of wild-type (WT) mice or tumor-bearing (TB) mice, the expression of TRAF6 in MDSCs from tumor tissue was significantly increased (Figures 1B,C). The expression of TRAF6 in PMN-MDSCs and M-MDSCs were determined by qRT-PCR, and the results showed that there was no difference in the mRNA expression of TRAF6 in the two subgroups of MDSCs (Figures 1D,E).

In addition, we analyzed the suppressive function of tumor tissue-derived MDSCs. Compared with MDSCs from spleen of TB mice, tumor tissue-derived MDSCs had stronger suppressive effects on the proliferation of CD4^+^ T cells (Figures 1F,G). Moreover, the expressions of Arg1 and iNOS in tumor tissue-derived MDSCs were higher than that in splenic MDSCs (Figures 1H,I). These results indicated that the immunosuppressive function of MDSCs derived from tumor tissue was stronger than those of splenic MDSCs from TB mice, which was consistent with previous reports (1).

### TRAF6 Knockdown Impairs the Immunosuppressive Activity of MDSCs *in vitro*

**Figure 2:**
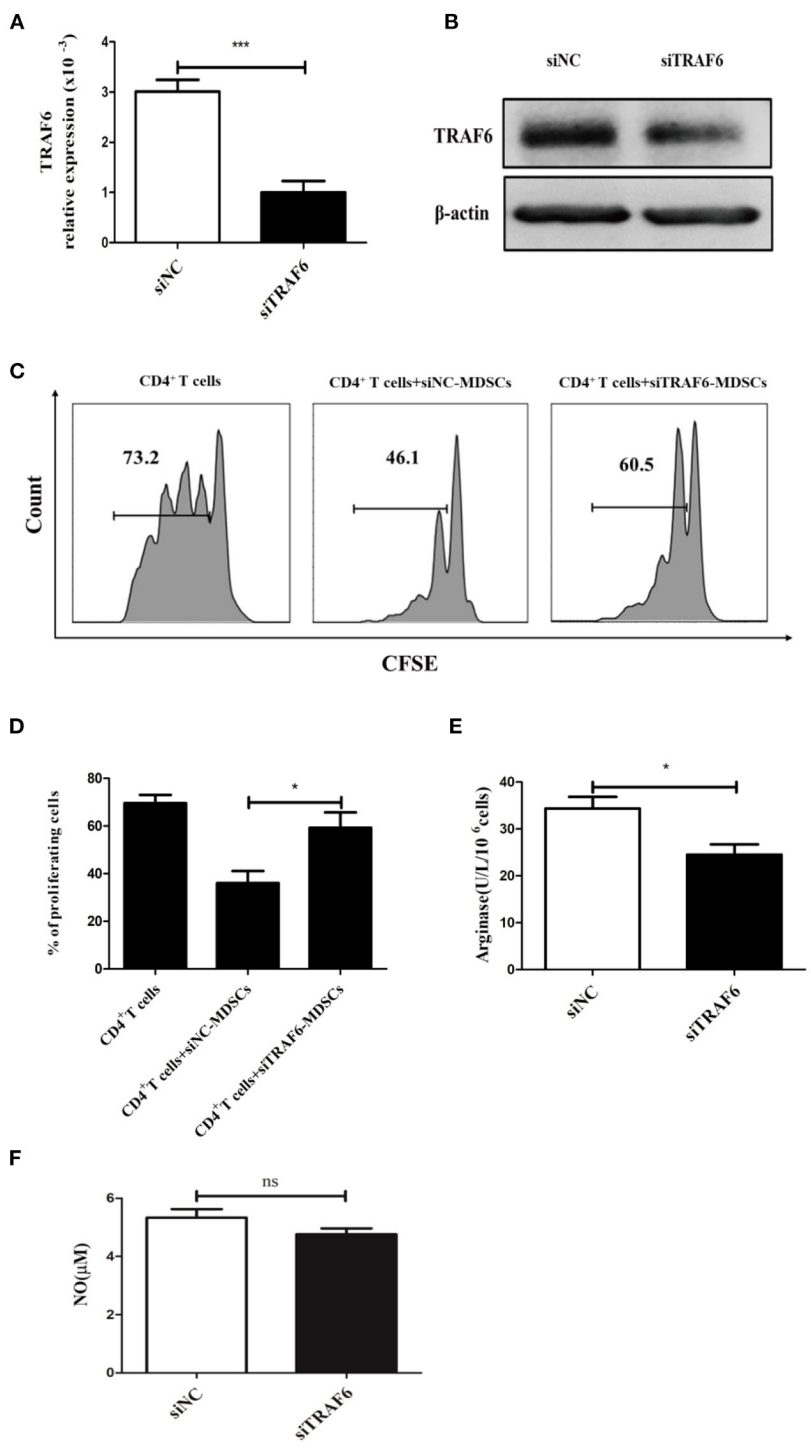
TRAF6 knockdown impairs the immunosuppressive effects of MDSCs *in vitro*. Specific siRNA (siTRAF6) was used to knockdown the expression of TRAF6 in MDSCs, and the efficiency of siTRAF6 knockdown was validated by qRT-PCR **(A)** and Western blotting **(B)**. **(C)** Tumor-derived MDSCs were transfected with siTRAF6 and cocultured with CFSE-labeled CD4^+^ T cells, and proliferation was measured by flow cytometry after 72 h. **(D)** Statistical analyses of the percentage of proliferating CD4^+^ T cells co-cultured with MDSCs transfected with siTRAF6. After TRAF6 knockdown, the activity of Arg1 was measured by a QuantiChrom arginase assay kit **(E)**, and the concentration of NO was determined via a Griess reagent system kit **(F)**. ***p < 0.001, *p < 0.05; ns, no significance.

To evaluate whether TRAF6 is involved in the suppressive effects of MDSCs, specific siRNA was used to knock down TRAF6 in MDSCs. After treatment with TRAF6-specific siRNA, the expression of TRAF6 in MDSCs from tumor tissue was effectively decreased (Figures 2A,B). Remarkably, the inhibitory effect of MDSCs transfected with siTRAF6 on CD4^+^ T cell proliferation was significantly attenuated (Figures 2C,D). Consistently, knockdown of TRAF6 distinctly decreased the activity of Arg1 in MDSCs (Figure 2E), although the content of NO did not noticeably altered (Figure 2F). These data indicate that knockdown of TRAF6 impairs the immunosuppressive effects of MDSCs *in vitro*.

### TRAF6 Alters the Activity of STAT3

**Figure 3:**
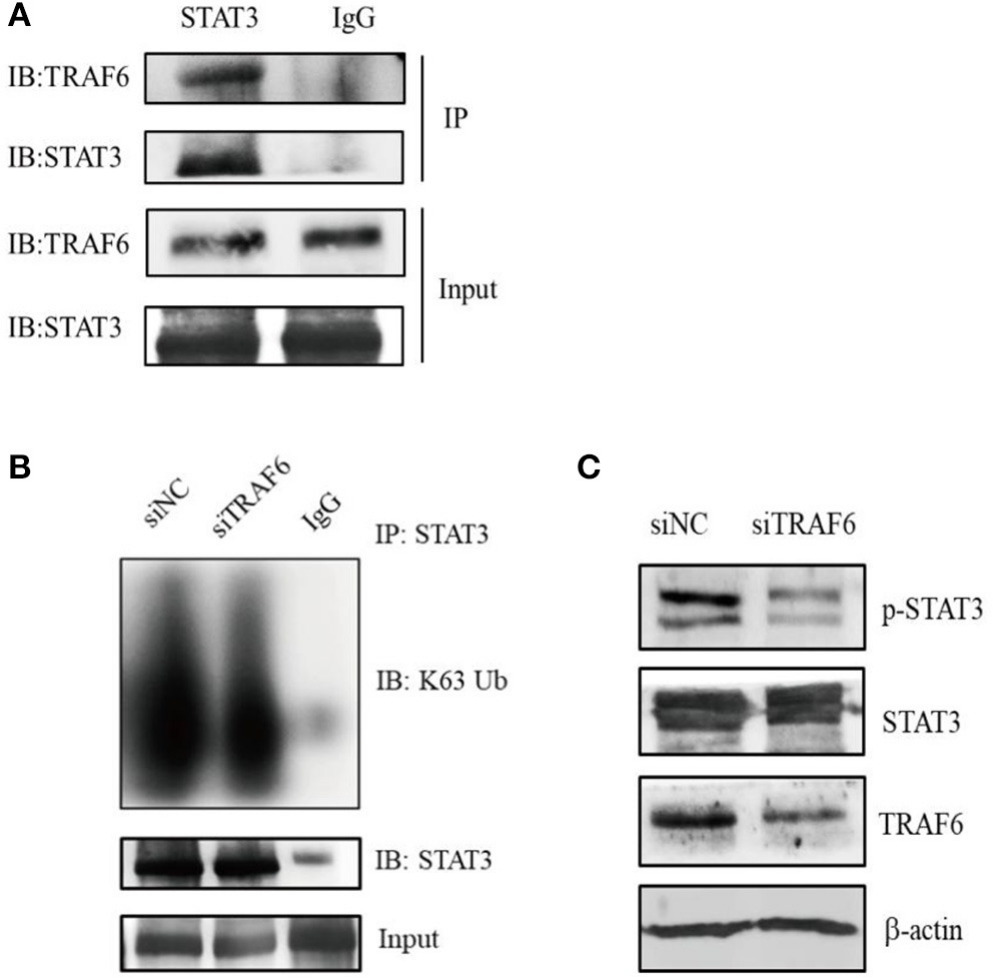
TRAF6 alters the activity of STAT3 by mediating the K63-linked polyubiquitination of STAT3 in MDSCs. **(A)** The interaction between TRAF6 and STAT3 in tumor-derived MDSCs was determined by co-immunoprecipitation (Co-IP) assays. **(B)** After knockdown of TRAF6 in MDSCs, the level of STAT3 K63-linked polyubiquitination was measured by Co-IP assays. **(C)** After knockdown of TRAF6 in MDSCs, the level of STAT3 phosphorylation was assessed by Western blotting.

Next, we explored the potential molecular mechanism by which TRAF6 regulates MDSC function. It is widely known that the transcription factor STAT3 plays a dominant role in MDSC expansion, activation and function. Recent studies have revealed that the E3 ubiquitin ligase TRAF6 mediates the K63-linked polyubiquitination of STAT3 but has no effect on STAT3 degradation (34). To determine whether TRAF6 impacts the function of MDSCs by STAT3, we conducted the interaction between TRAF6 and STAT3 in MDSCs by co-immunoprecipitation (Co-IP) assays. The results of Co-IP showed that TRAF6 was co-immunoprecipitated with STAT3, suggesting that endogenous TRAF6 binds to endogenous STAT3 in MDSCs (Figure 3A). Furthermore, we examined the regulation of TRAF6 on the posttranslational modification of STAT3. After knockdown of TRAF6 in MDSCs, the K63-linked polyubiquitination of STAT3 was significantly downregulated (Figure 3B). Moreover, silencing of TRAF6 remarkably decreased the levels of phosphorylated STAT3 in MDSCs from tumor tissue of TB mice (Figure 3C).

### TRAF6 Knockdown Attenuates the Ability of MDSCs to Accelerate Tumor Progression in Tumor-Bearing Mice

**Figure 4:**
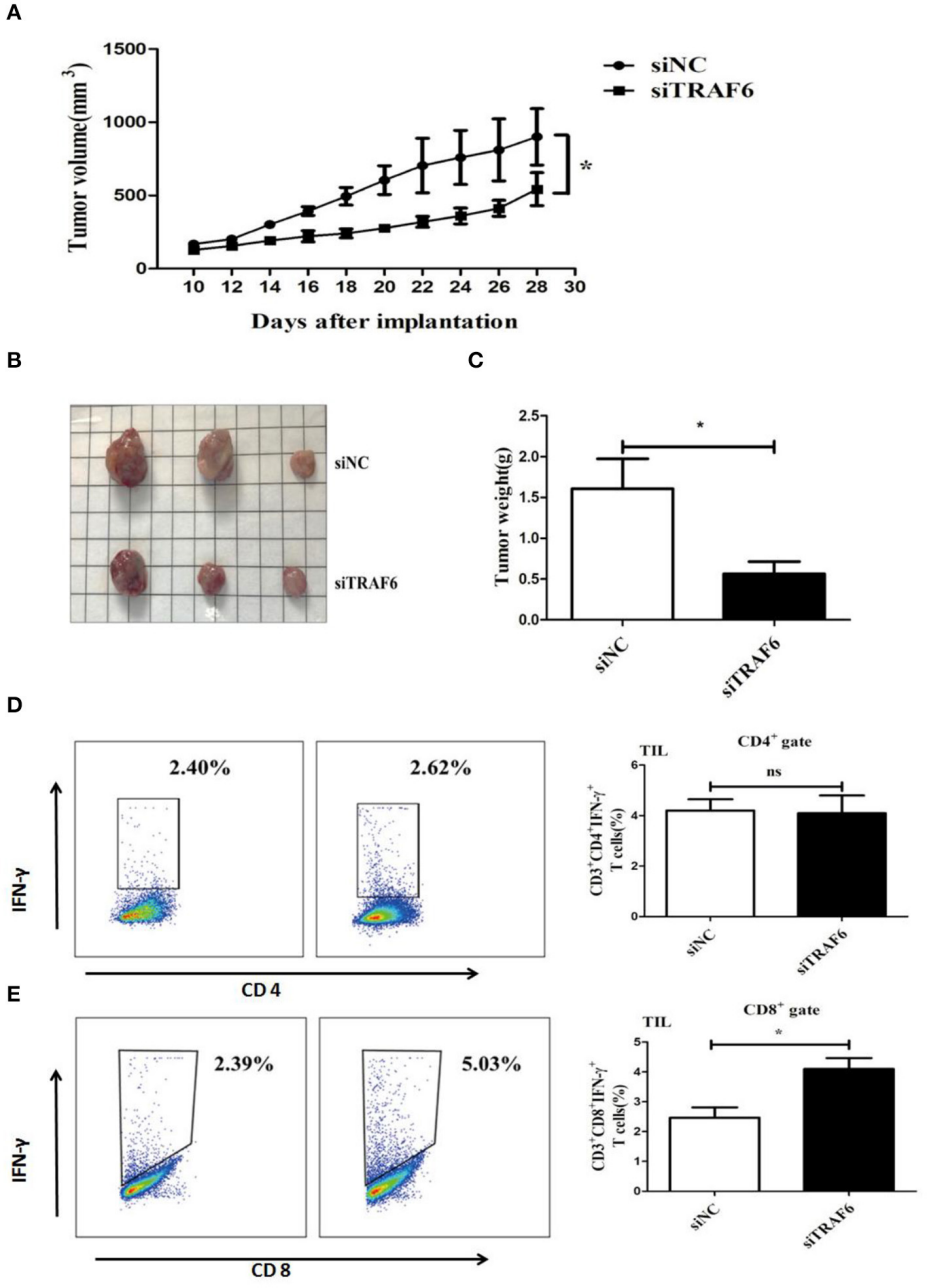
TRAF6 knockdown attenuates the ability of MDSCs to accelerate tumor progression in tumor-bearing mice. To investigate the effects of TRAF6 on the suppressive activity of MDSCs *in vivo*, 2 groups of wild-type C57BL/6 mice were s.c. injected with 1 × 10^6^ LLC cells and 1 × 10^6^ MDSCs transfected with siTRAF6 (siTRAF6 group) or MDSCs transfected with siNC (control group). **(A)** Tumor growth was constantly monitored. The width “a” and length “b” were measured, and tumor volume was calculated. **(B,C)** On the 28th day after the inoculation of LLC cells, the mice were sacrificed, and the tumor image and weights were showed in both groups. **(D)** The proportion of CD4^+^IFN-γ^+^ Th1 cells in the tumor tissue of both groups was analyzed by FCM. **(E)** The proportion of CD8^+^IFN-γ^+^ CTLs in the tumor tissue of both groups was analyzed by FCM. *p < 0.05; ns, no significance.

As shown in the *in vitro* experiments, knockdown of TRAF6 impaired the immunosuppressive activity of MDSCs. Thus, we further investigated the effects of TRAF6 on the suppressive effects of MDSCs *in vivo*. 1 × 10^6^ Lewis lung carcinoma cells and 1 × 10^6^ MDSCs transfected with TRAF6 siRNA or negative control siRNA were subcutaneously injected into C57BL/6 mice. As shown in Figure 4A, the tumor growth of mice injected with siTRAF6-transfected MDSCs (siTRAF6) group was evidently delayed. Furthermore, the tumor volume and weight were significantly less than those of the control (siNC) group (Figures 4B,C). Given that MDSCs mainly suppress the function of T cells in the tumor microenvironment, we measured the proportion of CD4^+^ Th1 cells and CD8^+^ CTLs from tumor tissue of TB mice. Compared with that of the control group, the proportion of Th1 cells was no significant difference in the siTRAF6 group (Figure 4D), while the proportion of CTLs in the siTRAF6 group was significantly increased (Figure 4E). Our data showed that knockdown of TRAF6 attenuated the ability of MDSCs to accelerate tumor progression and partly suppressed the antitumor T cell response.

### TRAF6 Expression Was Augmented in MDSCs From the PBMCs of Lung Cancer Patients

**Figure 5:**
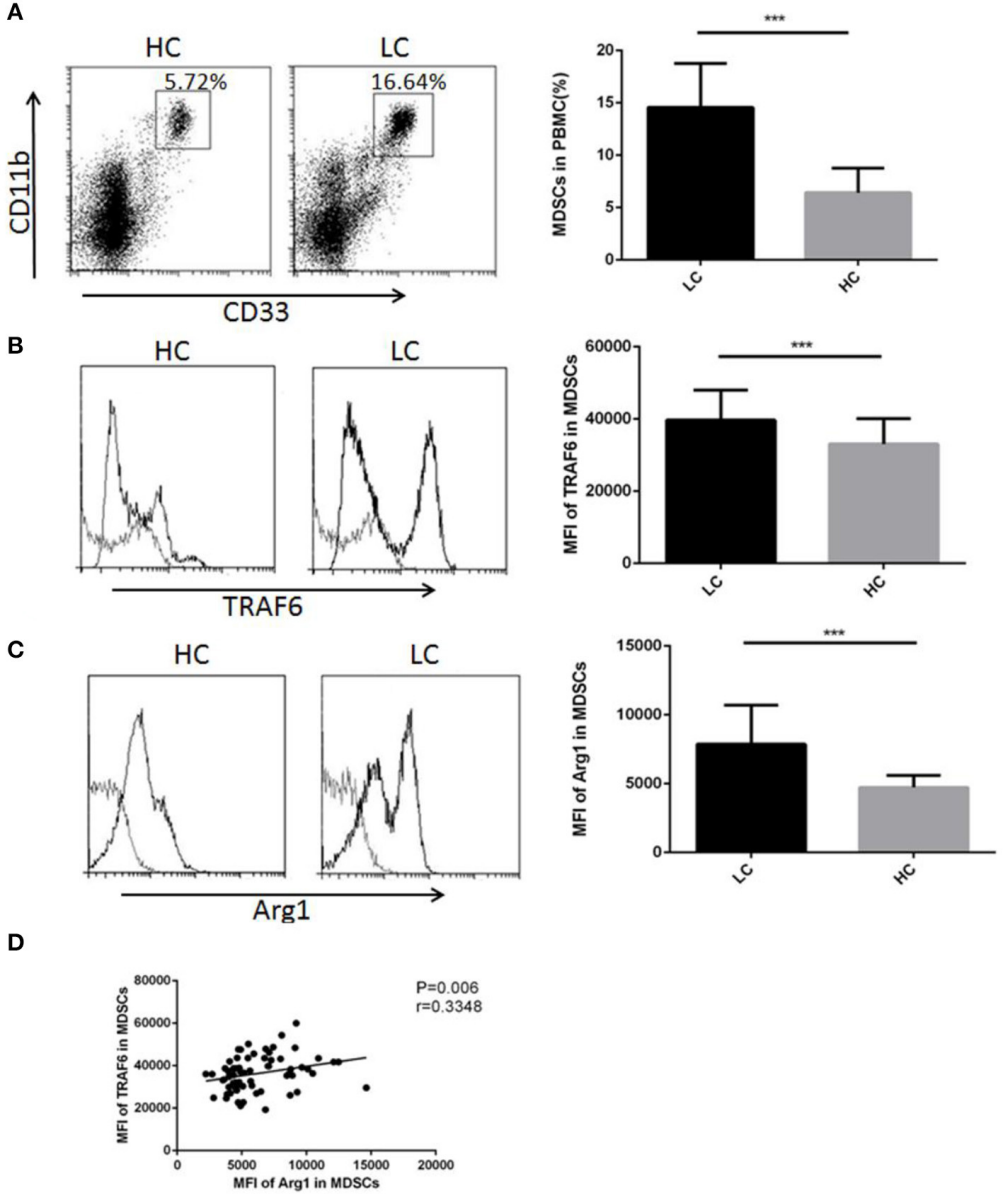
TRAF6 expression was augmented in MDSCs from lung cancer patients. To examine the modulation of TRAF6 in MDSCs from lung cancer patients, the level of TRAF6 in MDSCs was measured in the lung cancer patient group (LC) and healthy control group (HC). **(A)** The proportions of MDSCs in the PBMCs of lung cancer patients and healthy persons were analyzed by flow cytometry. Representative dot plots of CD11b^+^CD33^+^HLA-DR^−^ MDSCs in the blood of patients with LC and healthy controls are shown. **(B)** The mean fluorescence intensity (MFI) of TRAF6 in MDSCs was determined by flow cytometry. **(C)** The MFI of arginase-1 in MDSCs was determined by flow cytometry. **(D)** The correlation between TRAF6 and arginase-1 in MDSCs was analyzed. ***p < 0.001.

Considering that TRAF6 improves the function of MDSCs in mice, we examined whether TRAF6 had similar characteristics in MDSCs from lung cancer patients. We performed flow cytometry to analyze the proportion of CD11b^+^CD33^+^HLA-DR^−^ MDSCs in PBMCs from lung cancer patients. The percentage of MDSCs in PBMCs from lung cancer patients was higher than that from healthy controls, which indicated that the abnormal accumulation of MDSCs in peripheral blood was correlated with lung cancer (Figure 5A). We further examined the expression of TRAF6 in human MDSCs. Compared with that of healthy control, the expression of TRAF6 was markedly augmented in MDSCs from PBMCs of lung cancer patients (Figure 5B). In addition, Arg1 is the important immunosuppressive molecule in MDSCs, which was also upregulated in MDSCs from PBMCs of patients (Figure 5C). Furthermore, there was a positive correlation between the expression levels of TRAF6 and Arg1 (Figure 5D), indicating that TRAF6 may be participate in the regulation of Arg1 expression in MDSCs.

## Discussion

As an adaptor protein and E3 ubiquitin ligase, TRAF6 is involved in mediating various cellular signaling pathways and regulating a series of physiological process (35–40). In addition, the oncogenic role of TRAF6 in tumors has been widely reported. TRAF6 is abnormally highly expressed in multiple tumor tissues and modulates the malignant behavior of tumor cells (41–46). Furthermore, mounting evidence has revealed that TRAF6 plays a critical role in the development and activation of lymphocytes and myeloid cells (42, 47, 48). However, whether TRAF6 regulates MDSCs which are pivotal immunosuppressive cells has not been reported to date.

To shed light on the potential effect of TRAF6 on MDSC functions, we constructed murine Lewis lung carcinoma models. Our research suggested that the expression of TRAF6 was higher in MDSCs from tumor tissue than that in MDSCs from spleen. Consistent with previous research, we noted that the suppressive function of MDSCs derived from tumor tissue was stronger than that of spleen-derived MDSCs. Thus, we hypothesized that the strong suppressive activity of MDSCs from tumor tissues may be attributed to the high expression of TRAF6. After TRAF6 knockdown, the inhibitory effect of MDSCs on CD4^+^ T cell proliferation was significantly decreased, with reduced Arg1 activity. Moreover, knockdown of TRAF6 attenuated the ability of MDSCs to accelerate tumor progression in tumor-bearing mice. Many clinical studies have demonstrated that increased levels of both circulating and tumor-infiltrating MDSCs were associated with poor prognosis in cancer patients. We further confirmed that the percentage of MDSCs was markedly increased in lung cancer patients. In addition, the expression of TRAF6 was increased in MDSCs from lung cancer patients, which was positively correlated with the level of Arg1 in MDSCs. These results highlighted the key role of TRAF6 in enhancing the function of MDSCs *in vitro* and *in vivo*.

STAT3 is one of the most important transcription factors regulating the expansion, activation and function of MDSCs. STAT3 is abnormally activated in MDSCs in the tumor microenvironment (23, 24, 49, 50). In addition to phosphorylation, ubiquitination is also involved in STAT3 activation. In our study, we demonstrated that TRAF6 binds to STAT3 in MDSCs. TRAF6 knockdown markedly reduced the K63-linked polyubiquitination and phosphorylation of STAT3 in MDSCs, indicating that TRAF6 elevated the suppressive function of MDSCs by interacting with STAT3. Indeed, it has been reported that TRAF6 mediates K63 ubiquitination via the SH2 domain of STAT3, which is an essential step for STAT3 phosphorylation in response to bacterial infections (29). Thus we assumed that TRAF6 fosters the suppressive effect of MDSCs by inducing STAT3 K63 ubiquitination and subsequent STAT3 phosphorylation. However, further studies are required to reveal the detailed molecular mechanism by which TRAF6 affects the function of MDSCs via STAT3.

## Conclusions

In summary, we demonstrate that TRAF6 regulates the immunosuppressive activity of MDSCs by modulating the K63-linked polyubiquitination and phosphorylation of STAT3. Targeting of TRAF6 might be a potential clinical therapeutic strategy for enhancing antitumor immune response.

## Data Availability Statement

The original contributions presented in the study are included in the article/supplementary material, further inquiries can be directed to the corresponding author/s.

## Ethics Statement

The studies involving human participants were reviewed and approved by the Ethics Committee of the Affiliated People's Hospital of Jiangsu University. The patients/participants provided their written informed consent to participate in this study. The animal study was reviewed and approved by the Committee on the Use of Live Animals in Research and Teaching of Jiangsu University. Written informed consent was obtained from the individual(s) for the publication of any potentially identifiable images or data included in this article.

## Author Contributions

GS performed the experiments, analyzed the data, and wrote the paper. YZ analyzed the data, and wrote the paper. JT and JM analyzed the data. KY and HX revised the paper. SW designed the study and wrote the paper. All authors read and approved the final manuscript.

## Conflict of Interest

The authors declare that the research was conducted in the absence of any commercial or financial relationships that could be construed as a potential conflict of interest.

## Abbreviations

Arg1: Arginase 1
CTL: cytotoxic T lymphocyte
FCM: flow cytometry
iNOS: inducible nitric oxide synthase
IP: immunoprecipitation
MDSCs: myeloid-derived suppressor cells
M-MDSCs: monocytic myeloid-derived suppressor cells
NO: nitric oxide
PMN-MDSCs: polymorphonuclear myeloid-derived suppressor cells
ROS: reactive oxygen species
STAT3: signal transducer and activator of transcription 3
TRAF6: tumor necrosis factor receptor-associated factor 6
Ub: ubiquitin.
